# Generation of NBS1 knockout in Chinese hamster cells revealed ATR role for radiation and etoposide induced DNA damage in absence of NBS1 proteins

**DOI:** 10.3389/fonc.2026.1776137

**Published:** 2026-03-25

**Authors:** Gamze Badakul, Junko Maeda, Takamitsu A. Kato

**Affiliations:** Department of Environmental and Radiological Health Sciences, Colorado State University, Fort Collins, CO, United States

**Keywords:** ATR, cell survival, Chinese hamster, CRISPR/Cas9, NBS1

## Abstract

Nijmegen Breakage Syndrome (NBS) is a rare autosomal recessive disorder characterized by chromosomal instability, immunodeficiency, radiosensitivity, and a strong predisposition to lymphoid malignancies. It is caused by mutations in the NBN gene encoding nibrin (NBS1) protein, a core component of the MRE11-RAD50-NBS1 (MRN) complex that senses DNA double-strand breaks (DSBs) and coordinates DNA damage response, including ATM activation. Despite the importance of NBS1, in the Chinese hamster system, which offers significant advantages in radiation biology and toxicology, no mutant lines deficient in the NBS1 gene have been isolated. In this study, we generated two novel NBS1 mutant Chinese hamster cell lines using CRISPR/Cas9, each carrying distinct NBN mutations leading to either null or hypomorphic mutations. These mutants exhibited growth retardation, marked sensitivity to ionizing radiation and various DNA damaging agents and elevated radiation induced chromosomal aberrations, recapitulating key NBS phenotypes. Notably, NBS1 mutant cells displayed pronounced hypersensitivity to ionizing radiation when co-treated with an ATR inhibitor, but not with a DNA-PK inhibitor. The ATR inhibitor also markedly sensitized NBS1 mutants to Etoposide, suggesting that ATR functions as a compensatory pathway in the absence of functional NBS1 during specific types of DNA damage. Collectively, our findings establish valuable NBS1-deficient Chinese hamster cell models that expand understanding of NBS1 function and highlight their utility for investigating DNA repair deficiencies and developing targeted therapeutic approaches for chromosomal instability disorders and cancers with NBS1 mutations.

## 1 Introduction

Nijmegen Breakage Syndrome (NBS) is a rare autosomal recessive chromosomal instability disorder characterized by microcephaly, growth retardation, immunodeficiency, and a marked predisposition to lymphoid malignancies (1). The disease is caused by mutations in the NBN gene, which encodes nibrin (NBS1), a key regulator of the DNA damage response (DDR) (2). NBS1 plays a central role in the cellular response to DNA damage, particularly in the repair of DNA double strand breaks (DSBs). Loss or mutation of NBS1 results in defective DDR signaling and pronounced hypersensitivity to ionizing radiation (3, 4).

NBS1 is an essential component of the MRE11-Rad50-NBS1 (MRN) complex, which functions in detecting DSBs, activation of ATM kinase, and coordination of DSB repair pathway choice between homologous recombination (HR) and non-homologous end joining (NHEJ) (5, 6). In addition to its canonical role in DSB repair, NBS1 has been implicated in the regulation of cell cycle checkpoints, telomere maintenance, apoptosis, and replication stress responses (7–9). Somatic alterations in NBN have also been identified in multiple sporadic tumors (10), suggesting that NBS1 exerts broader tumor suppressive functions beyond the context of inherited NBS.

In addition to patient-derived cell lines, a variety of experimental models have been used to investigate NBS1 function, including mouse Nbs1-null (11), the human breast cancer cell line HCC1935 (10), and Nbs1-knockout DT40 chicken cells (5). These cell lines demonstrated MRN-dependent checkpoint functions and replication-stress responses (9, 12). Together, these systems highlight conserved MRN functions and revealed context-dependent consequences of NBS1 deficiency.

Although human cells derived from NBS patients are available, their limited proliferative capacity, poor plating efficiency, and genetic heterogeneity restrict their utility for mechanistic and therapeutic studies. In contrast, Chinese hamster isogenic cell models offer distinct advantages, including rapid growth, high cloning efficiency, and a stable karyotype well suited for cytogenetic and radiation biology analyses. Expanding the Chinese hamster mutant cell line panel to include NBS1-deficient models will provide a powerful experimental platform for investigating cellular responses to ionizing radiation and genotoxic stress and for evaluating therapeutic vulnerabilities associated with NBS1 loss.

In this study, we utilized the CRISPR/Cas9 system to generate NBS1-deficient Chinese hamster cell lines and systematically characterized their DDR phenotypes. We identify a unique ATR inhibition-driven sensitization of NBS1-deficient cells to ionizing radiation and the topoisomerase II inhibitor etoposide, revealing a critical compensatory role for ATR signaling in the absence of functional NBS1. These findings provide new insight into genome maintenance mechanisms in NBS and highlight potential therapeutic strategies for targeting NBN-deficient malignancies.

## 2 Materials and method

### 2.1 Cell culture

Chinese hamster lung-origin V79 cells were generously provided by Dr. Joel Bedford (Colorado State University, Fort Collins, CO, USA). Cells were cultured in Alpha Minimum Essential Medium (Alpha-MEM; Thermo Fisher Scientific, Waltham, MA, USA) supplemented with 10% heat-inactivated fetal bovine serum (FBS; Sigma-Aldrich, St. Louis, MO, USA) and 1% antibiotic–antimycotic solution (Anti-Anti; Invitrogen, Grand Island, NY, USA). Cultures were maintained at 37 °C in a humidified incubator with 5% CO_2_.

### 2.2 Chemicals

The PARP inhibitor olaparib was obtained from Calbiochem (San Diego, CA, USA). The topoisomerase I inhibitor camptothecin, topoisomerase II inhibitor etoposide, DNA replication inhibitor hydroxyurea, DNA crosslinking agent cisplatin, alkylating agent methyl methanesulfonate (MMS), and DNA double-strand break (DSB)-inducing agent bleomycin were all purchased from Sigma-Aldrich. The ATM inhibitor KU55933 (13), ATR inhibitor VE-821 (14), and DNA-PK inhibitor NU7441 (15) were obtained from Selleck Chemicals (Houston, TX, USA). The DNA crosslinking agent Mitomycin C (MMC) was purchased from Funakoshi (Tokyo, Japan).

### 2.3 Irradiation

Gamma irradiation was performed at room temperature using a ^137^Cs gamma-ray irradiator (Model Mark I-68, 6000 Ci; J.L. Shepherd, Carlsbad, CA, USA) at a dose rate of 2.5 Gy/min. UV-C irradiation was carried out using Philips germicidal lamps (Philips, Andover, MA, USA) at a fluence rate of 1 W/m², as previously described (16).

### 2.4 CRISPR-Cas9 gene editing and cloning of knockout cells

**Figure 1:**
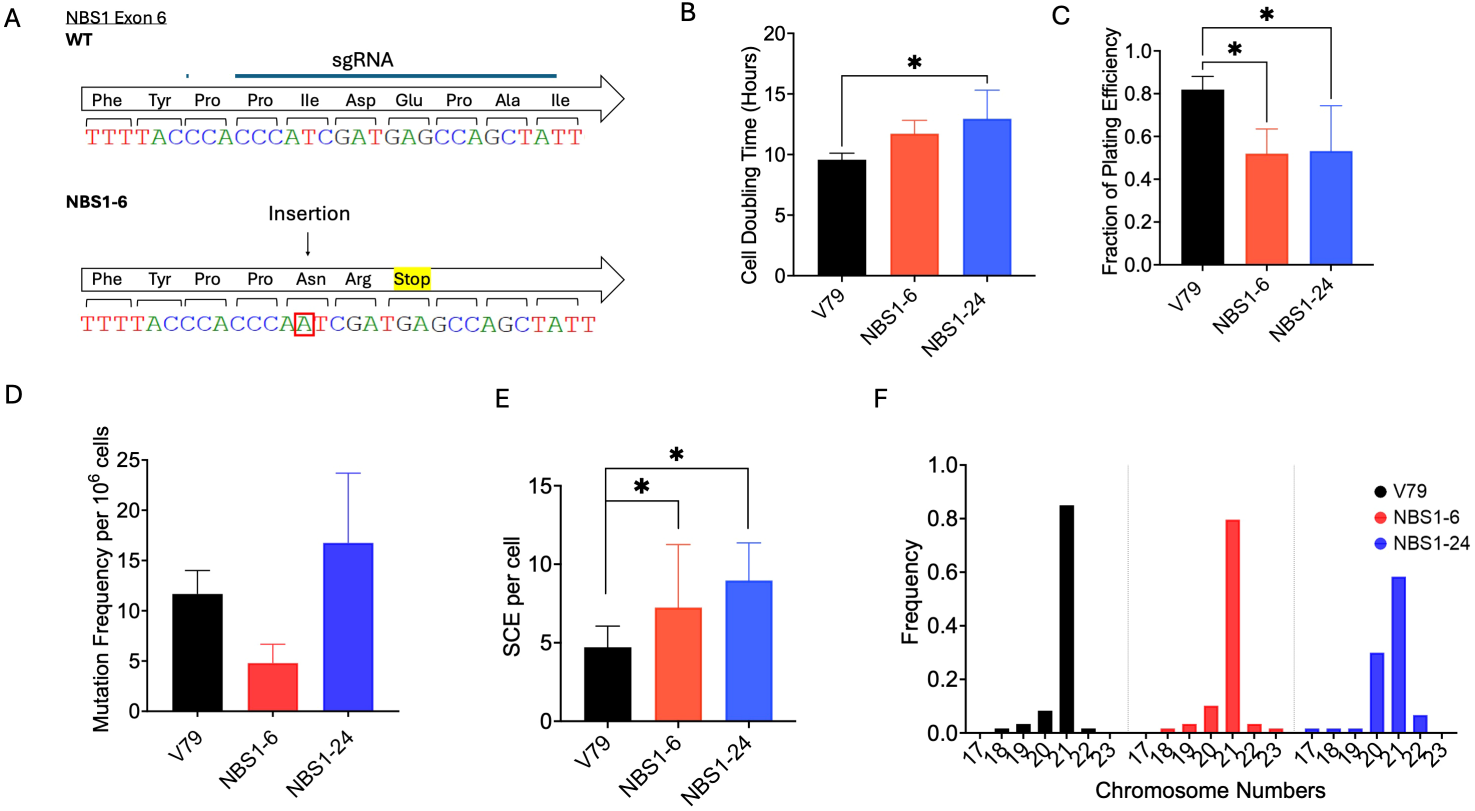
Characterization of two NBS1 mutant cell lines. **(A)** Sanger sequence result of the NBS1 mutant clone NBS1-6. **(B)** Cell doubling time. **(C)** Plating efficiency. **(D)** HPRT mutation frequency analysis. **(E)** Sister chromatid exchange (SCE). **(F)** Chromosome numbers. Error bars indicate the standard error of the mean from at least three independent experiments. *indicates statistical significance (p<0.05, ANOVA).

Single guide RNAs (sgRNAs) targeting the Chinese hamster NBS1 gene were designed using the CHOPCHOP online tool (http://chopchop.cbu.uib.no), referencing the CHO genome assembly GCF_003668045.3 as previously described (17). sgRNAs were selected for high targeting efficiency and minimal predicted off-target effects. sgRNAs near the N-terminus were prioritized to ensure potential expression of partially functional truncated proteins if functional domains were affected. One day prior to transfection, V79 cells were seeded at 1.2 × 10^5^ cells per 35 mm dish in 2 mL of culture medium. Cells were transfected with CRISPR/eSpCas9 plasmids (GenScript, Piscataway, NJ, USA) expressing sgRNA targeting exon 6 of NBS1 (sequence: ATAGCTGGCTCATCGATGGG) using Lipofectamine 2000 (Invitrogen) in Opti-MEM, and incubated for 48 hours. After six days of selection with 8 μg/mL puromycin, single-cell clones were obtained by serial dilution in 96-well plates and visually selected. A total of 24 single cell derived clones were initially screened for NBS1 deficiency by sensitivity to camptothecin (5 nM). Genomic DNA was extracted from camptothecin-sensitive clones, and the target region within exon 6 of NBS1 was amplified by PCR using the forward primer GCCAGATTGGCCTTGAACTTTT and reverse primer ACATGCAGACAAAACACCCAC. RT-PCR was performed using forward primer CAGCTTGGAGGACTTACAGCA and the reverse primer TTCTTCTGCCATCAGCCTGG. PCR products were analyzed by Sanger sequencing to confirm CRISPR-Cas9-mediated mutations (**Figure 1A**). Potential off-target effects were minimized by selecting sgRNAs with high predicted specificity using the CHOPCHOP web tool and checked the list of the candidate off-target sites if editing at those sites would not confound NBS1 function; therefore, genome-wide off-target analyses were not performed.

For protein analysis, selected clones were lysed using M-PER Mammalian Protein Extraction Reagent (Thermo Fisher Scientific, Waltham, MA, USA) supplemented with protease inhibitors. Protein concentrations were determined, and 50 µg of total protein per sample was subjected to SDS–PAGE at 80 V for 1 hour, followed by transfer to a PVDF membrane. After blocking for 1 hour at room temperature, membranes were incubated with rabbit anti-p95/NBS1 primary antibody (1:250 dilution; Cat#FO868, Selleckchem, Houston, TX, USA), followed by goat anti-rabbit IgG HRP-conjugated secondary antibody (1:1000 dilution; Cell Signaling Technology, Danvers, MA, USA). Protein signals were detected using SuperSignal West Femto chemiluminescent substrate (Thermo Fisher Scientific) and visualized using a Bio-Rad ChemiDoc imaging system. Membranes were then stripped and applied for beta-actin mouse antibody (1:1000 dilution; Abcam, ab8226) followed by goat anti-mouse IgG HRP conjugated secondary antibody (1:3000 dilution; Abcam ab97023).

### 2.5 HPRT mutation frequency

Cells were cultured in Alpha Minimum Essential Medium (Alpha-MEM) supplemented with 10% heat-inactivated fetal bovine serum (FBS) and 1% antibiotic–antimycotic solution. After several days, all cell lines were transferred to DF Medium [Dulbecco’s Modified Eagle’s Medium/Ham’s F-12 (1:1)] supplemented with 10% FBS. HPRT mutants were selected using 6-TG Medium, consisting of DF Medium without hypoxanthine and supplemented with 40 µM (6.7 µg/mL) 6-thioguanine (6-TG) (Sigma-Aldrich, St. Louis, MO). To eliminate pre-existing HPRT^-^ mutants, cells were pretreated with HAT Medium [DF Medium containing 100 µM hypoxanthine, 0.4 µM aminopterin, and 16 µM thymidine] (Sigma-Aldrich). The initial frequency of HPRT^-^ cells was determined in all cell lines. A total of 1 × 10^6^ cells were plated in 6-TG Medium at a density of 5 × 10^4^ cells per 10-cm dish. In parallel, 5 × 10² cells per 10-cm dish were plated in triplicate in DF Medium (without hypoxanthine) to assess plating efficiency (PE) at the time of selection. Cells were incubated for 12–14 days, and colonies were visualized by staining with 0.5% crystal violet (Sigma-Aldrich; in 50% methanol, v/v). The number of 6-TG–resistant (6-TG^r^) colonies and the PE were used to calculate mutant frequency using the following equation: MF=a/(10^6^ x [b/1.5x10^3^]), where a = total number of 6-TG^r^ colonies and b = total number of colonies on all 3 PE plates.

### 2.6 Sister chromatid exchange analysis

After mitotic shake-off, cells were supplemented with 10 µM BrdU. Following a 20-hour incubation, colcemid was added for 6 hours to arrest cells in metaphase. Metaphase chromosome spreads were then prepared for chromosome aberration analysis. Slides were incubated with 5 µg/mL Hoechst 33258 for 15 minutes, exposed to UVB for 15 minutes, and treated with 2× SSC at 80 °C for 15 minutes as previously described (18). Cells were stained with 5% Giemsa. The number of sister chromatid exchange (SCE) was scored using a Zeiss Axioskop microscope. For each experiment, at least 50 metaphase spreads were evaluated for SCE scoring.

### 2.7 Colony formation assay

Cellular sensitivity to radiation or chemotherapeutic agents was evaluated using colony formation assays. Cells were plated and treated with various doses of test agents. After seven days of incubation, colonies were fixed with ethanol and stained with crystal violet. Colonies containing >50 cells were considered survivors as previously described (17). Survival curves were generated, and IC_50_ or D_10_ values were calculated using GraphPad Prism 8 from at least three independent experiments.

### 2.8 G2 premature chromosome condensation assay

Exponentially growing cells were irradiated and treated with 50 nM Calyculin A (Sigma-Aldrich) for 30 minutes. Chromosome spreads were prepared and scored for chromatid aberrations, including gaps, breaks, iso-breaks, and exchanges, using a Zeiss Axioskop microscope. A minimum of 50 premature chromosome condensation (PCC) spreads were analyzed in three independent experiments.

### 2.9 G2/M checkpoint arrest and S/G2 chromosome aberrations

G2 chromosomal radiosensitivity was evaluated in G2 assay as previously described (17). Log-phase cells were irradiated with 1 Gy and incubated for 30 minutes, followed by the addition of 0.1 μg/mL colcemid for 1.5 hours. All cells including mitotic cells were collected, treated with hypotonic KCl, and fixed in methanol: acetic acid (3:1). Metaphase spreads were prepared, stained with 5% Giemsa, and analyzed using a Zeiss Axioskop microscope. Mitotic index was determined from at least 100 cells, and chromatid breaks and exchanges were scored in 50 metaphase cells per experiment, across three independent replicates.

To assess S/G2 chromosomal sensitivity, cells were plated and treated with camptothecin (5 nM) and VE-821 (1 μM) for 3 hours, followed by the addition of Colcemid (0.1 μg/mL) for 3 hours to arrest cells in metaphase. For etoposide, cells were treated with etoposide (1 nM) and VE-821 (1 μM) and Colcemid (0.1 μg/mL) for 3 hours. After treatment, all cells were collected, processed and evaluated for G2 chromosomal aberrations as described above.

### 2.10 Immunofluorescence analysis for DNA damage responses

Following overnight drug treatment cells were fixed with 4% paraformaldehyde for 15 minutes and permeabilized with 0.5% Triton X-100 for 10 minutes. After blocking with 10% goat serum in PBS for 30 minutes, cells were incubated with primary antibodies against Rad51(D4B10) (#88755, Cell Signaling Technology, Danvers, MA, USA) and γH2AX (NG1-904671, Sigma-Aldrich). Secondary antibodies used were Alexa Fluor 488-conjugated goat anti-mouse and Alexa Fluor 594-conjugated goat anti-rabbit (Invitrogen). Coverslips were mounted using SlowFade with DAPI (Invitrogen), and images were captured using a Zeiss Axioskop fluorescence microscope equipped with a motorized z-stage and CoolSNAP HQ2 camera and Metamorph software (Molecular Devices, San Jose, CA, USA).

### 2.11 Flow cytometry analysis

Exponentially growing cells were exposed to drugs overnight and fixed in 70% ethanol. Cells were treated with 0.5 mg/ml RNase A and stained with 20 μg/ml propidium iodide. Flow cytometry analysis was conducted with Dako Cyan ADP flow cytometer with Summit software (Beckman Coulter, Indianapolis, IN). At least 10,000 cells were analyzed, and cell cycle distribution was analyzed with ModFIt LT software (Verity Software, Lexington, MA).

### 2.12 Statistical analysis

All experiments were performed in triplicate or more with biologically independent samples. Data are expressed as mean ± standard error of the mean (SEM). Statistical significance was evaluated using one-way ANOVA and two-way ANOVA in GraphPad Prism 8. Differences with p-values < 0.05 were considered statistically significant.

## 3 Results

### 3.1 Characterization of isolated clones

To generate NBS1 mutant cells using the CRISPR/Cas9 system, sgRNA targeting exon 6 of the NBS1 gene in the Chinese hamster genome were employed. After transfection, two clones were obtained. Sequence analysis has revealed that NBS1–6 and NBS1–24 have different types of mutations in exon 6. In NBS1-6, a single-base insertion of adenine resulted in a frameshift mutation predicted to substitute two amino acids followed by a premature stop codon [XM_027403560.2, c.599_600insA, p.(Ile200Asnfs*3)] (**Figure 1A**). This truncation is expected to disrupt downstream functional domains critical for MRN complex stability and ATM-dependent signaling.

In NBS1-24, a 80 bp deletion spanning intron 5 and exon 6 was identified by genomic PCR. RT-PCRidentified three transcripts (**Supplementary Figure 1**). Transcript analysis by RT-PCR demonstrated that the shortest band with 248 bp amplification was exon 6 skipping and caused frameshift mutation with premature stop site. Two larger bands indicated large insertion containing part of the Cas9 sequences (pX459V2.0eSpCas9, Sequence ID: LC740575.1).

Western blot analysis detected an NBS1 protein of smaller size in V79 cells (**Supplementary Figure 2**). No detectable NBS1 proteins for NBS1-6, whereas NBS1–24 expressed a larger NBS1 protein consistent with altered splicing. Based on these molecular findings, NBS1–6 is classified as a null mutant, while NBS1–24 represents a hypomorphic mutant with abnormal but detectable NBS1 protein expression.

Two NBS1 mutant cells displayed growth and proliferation retardation. The doubling times of the mutant cell lines ranged from 11.7 to 13 hours (**Figure 1B**). NBS1–24 displayed a significantly reduced growth rate compared with the wild type (p < 0.05), whereas NBS1–6 did not differ significantly from V79 cells. Plating efficiencies were 82.9% for V79, whereas 52% for NBS1-6, and 53% for NBS1-24, with statistically significant differences (**Figure 1C**). Two NBS1 mutants were not hyper-mutagenic as observed in HPRT mutation frequency assays with no significant differences compared to wild type cells (**Figure 1D**). On the other hand, sister chromatid exchange (SCE) analysis showed a significant increase in mutants compared with the wild type (p < 0.05) (**Figure 1E**). Chromosome number analysis indicated that, although the NBS1–24 exhibited additional numerical variants, the modal chromosome number remained unchanged at 21, consistent with wild-type cells (**Figure 1F**).

### 3.2 Cytotoxicity against various DNA-damaging agents

**Figure 2:**
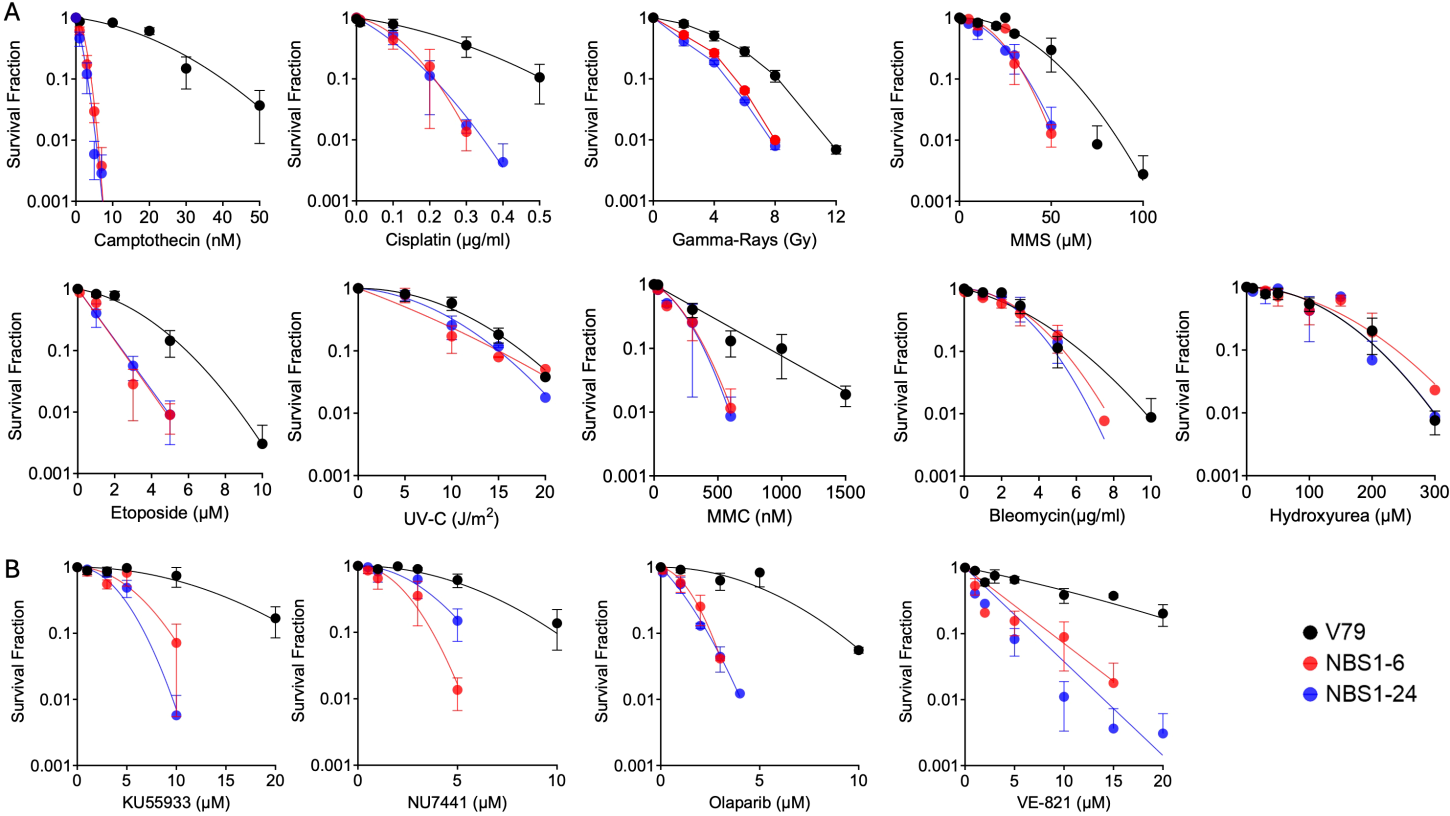
Sensitivity to DNA damage response agents and inhibitors. **(A)** Cytotoxicity to various DNA-damaging agents including bleomycin, MMS, MMC, hydroxyurea, cisplatin, etoposide, camptothecin, ionizing radiation, and UV-C. **(B)** Cytotoxicity to DNA repair inhibitors, including KU55933 (ATM inhibitor), NU7441 (DNA-PK inhibitor), Olaparib (PARP inhibitor), and VE-821 (ATR inhibitor). Error bars indicate the standard error of the mean from three independent experiments.

Given the role of NBS1 in DNA damage response, we assessed cell survival following exposure to ionizing radiation, UV-C, and various genotoxic agents, including bleomycin, camptothecin, cisplatin, etoposide, hydroxyurea, mitomycin C and methyl methane sulfonate (MMS), using colony formation assays (**Figure 2A**). NBS1 mutants displayed hypersensitivity to all tested agents except bleomycin and hydroxyurea. NBS1 mutations increased sensitivity to particularly camptothecin (topoisomerase I inhibitor) and etoposide (topoisomerase II inhibitor) with the reduction of IC_50_ values more than three times. Summary cytotoxicity data are presented in **Table 1**.

**Table 1:** **A** summary of D_10_ and IC_50_ values (mean ± SEM) for DNA-damaging agents.

Treatment	V79	NBS-6	NBS1-24
Gamma-ray (Gy)	8.2 ± 0.4	5.6 ± 0.2 *	4.7 ± 0.2 *
Camptothecin (nM)	17.0 ± 1.8	1.9 ± 0.2 *	0.9 ± 0.2 *
Cisplatin (µg/ml)	0.22 ± 0.04	0.10 ± 0.02 *	0.09 ± 0.02 *
MMS (μM)	28.0 ± 3.6	15.2 ± 4.2	16.1 ± 3.6
UV-C (J/m^2^)	19.5 ± 1.3	14.6 ± 0.8	14.6 ± 0.9
Etoposide (μM)	2.1 ± 0.4	0.6 ± 0.1 *	1.1 ± 0.1 *
Mitomycin C (nM)	259.5 ± 57.6	166.2 ± 40.7	128.9 ± 34.7
Bleomycin (µg/ml)	2.41 ± 0.4	2.4 ± 0.7	2.6 ± 0.8
Hydroxyurea (μM)	93.6 ± 13.1	88.7 ± 26.2	122.6 ± 51.5
KU55933 (μM)	10.6 ± 2.2	4.2 ± 0.4 *	3.1 ± 0.2 *
NU7441 (μM)	4.7 ± 1.3	1.9 ± 0.4	2.8 ± 0.8
Olaparib (μM)	4.0 ± 0.5	1.1 ± 0.3 *	0.9 ± 0.2 *
VE-821 (μM)	8.9 ± 1.3	3.0 ± 0.8 *	2.0 ± 0.2 *

*indicates mutant cells displayed more than three times differences in D_10_ or IC_50_ values compared to wild type.

### 3.3 Cytotoxicity against DNA damage response inhibitors

Elevation of cytotoxicity to DNA damage response (DDR) inhibitors targeting ATM, ATR, DNA-PK, and PARP was assessed in NBS1 mutants using colony formation assays. Cells were treated with KU55933 (ATM inhibitor), NU7441 (DNA-PKcs inhibitor), Olaparib (PARP inhibitor), and VE-821 (ATR inhibitor). Both NBS1–6 and NBS1–24 clones exhibited hypersensitivity to all tested inhibitors compared with wild-type V79 cells (**Figure 2B**), indicating that those DDR proteins’ activities are necessary in NBS1 mutants, and loss of NBS1 compromises multiple DNA repair pathways. Among tested four inhibitors, Olaparib and VE-821 have shown severe cytotoxicity to NBS1 mutant cells as observed with more than 2 times reduction of IC_50_ values (**Table 1**).

### 3.4 Radiation induced DNA damage responses in NBS1 mutant cells

**Figure 3:**
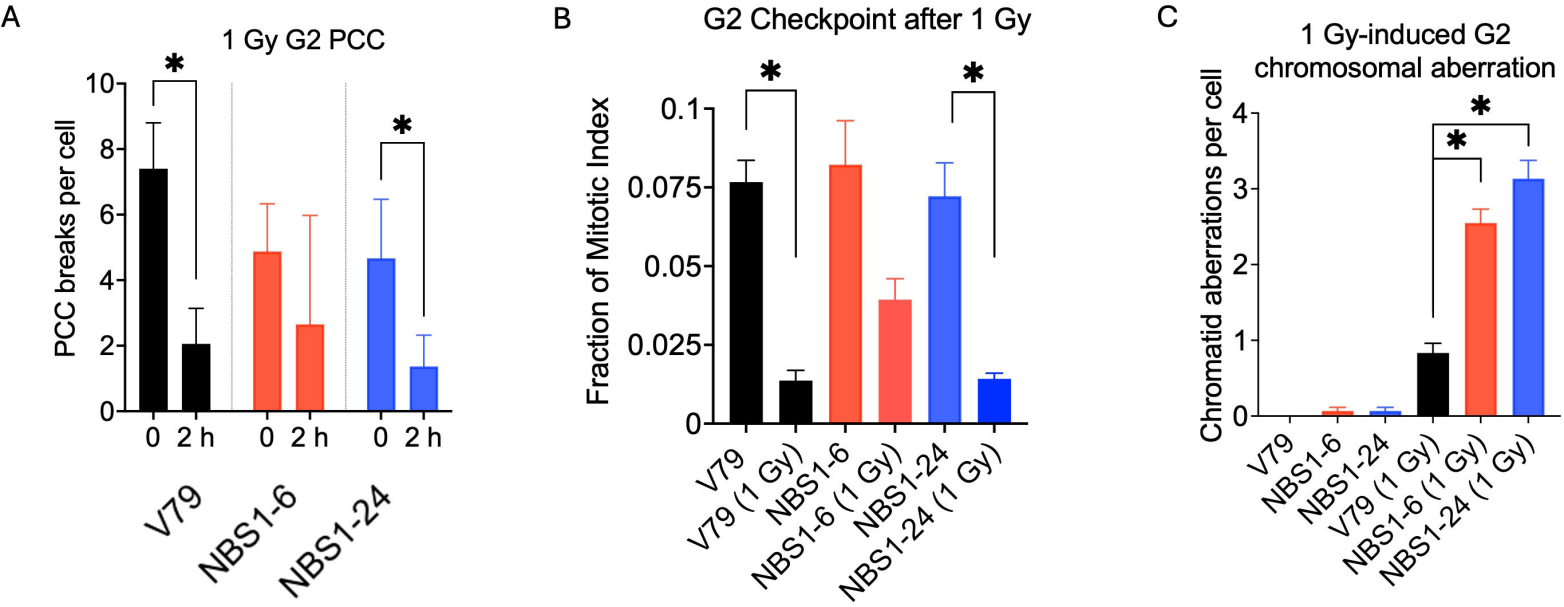
Radiation induces DNA damage responses (DDRs) in NBS1 mutant cells. **(A)** Chromatin break and repair analysis by G2 premature chromosome condensation (PCC), **(B)** Mitotic index analysis after G2-assay, and **(C)** Chromosomal aberration analysis after G2-assay after 1 Gy of irradiation. Error bars indicate the standard error of the mean from three independent experiments. *indicates statistical significance (p<0.05, ANOVA).

Chromatin break repair during G2 was analyzed by Calyculin A induced premature chromosome condensation (PCC) assay (**Figure 3A**). G2 PCC breaks were analyzed at 0 and 2 hours after 1 Gy irradiation. The initial average PCC breaks were 7.4 (V79), 4.9 (NBS1-6) and 4.7 (NBS1-24) among three cell lines. On the other hand, the V79 and NBS1–24 cells showed a significantly reduced number of G2 PCC breaks after 2 hours of irradiation whereas there is no significant reduction for NBS1 null NBS1–6 cells.

G2-assay was conducted to evaluate radiation induced G2/M arrest and chromosomal aberration formation. Without irradiation, treatment of colcemid displayed mitotic index of 0.073-0.083 for three cell lines without statistically significant differences. With 1 Gy of irradiation, V79 cells presented significant reduction of the mitotic index to 0.014. Two NBS-1 mutants displayed mixed results between NBS1–6 and NBS1-24. Although NBS1–6 displayed minimum G2/M arrest with mitotic index of 0.04 without statiscally significant reduction from unirradiated control, mitotic index of NBS1–24 was 0.014 with significant reduction (**Figure 3B**). In contrast, chromosomal aberrations observed as chromatid breaks were greatly induced in both NBS1 mutant cells. Although spontaneous level chromatid breaks were comparable among wild type and the two NBS1 mutant cells, NBS1 mutant cells displayed approximately three times more chromatid type aberrations in NBS1–6 and four times more in NBS1–24 after 1 Gy of irradiation (**Figure 3C**).

### 3.5 Combinations of DDR inhibitors and ionizing radiation, camptothecin, and etoposide

**Figure 4:**
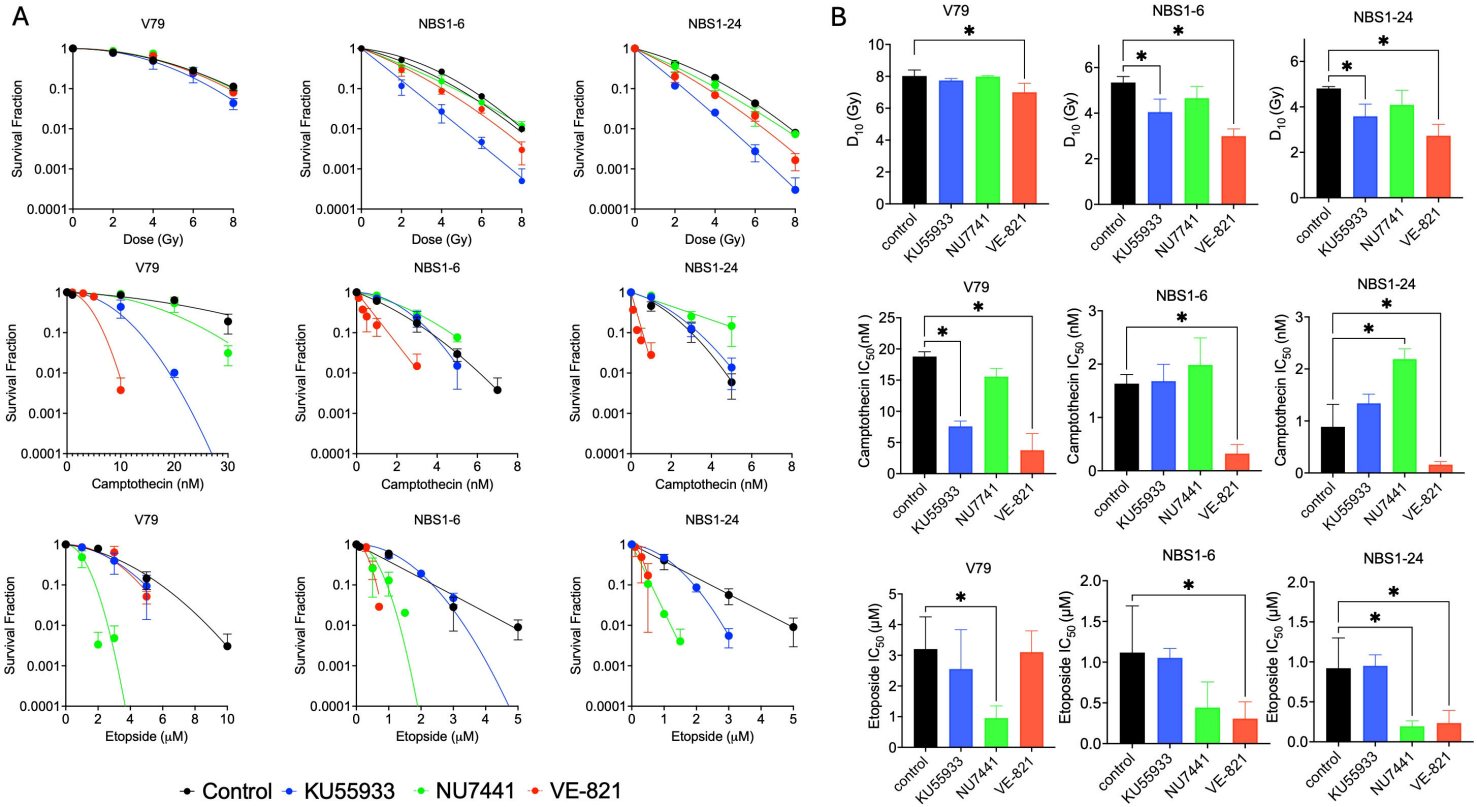
Combination of DNA Damage agents with DDR inhibitor in NBS1 mutant cells. **(A)** Cytotoxicity in combination with ionizing radiation, camptothecin, and etoposide against ATM, ATR, and DNA-PK inhibitors. **(B)** D_10_ and IC_50_ values obtained from the cell survival curves. Error bars indicate the standard error of the mean from three independent experiments. *indicates statistical significance (p<0.05, ANOVA).

To assess functional redundancy between NBS1 and the DDR kinases ATM, ATR, and DNA-PK, cells were treated with specific DDR kinase inhibitors in combination with ionizing radiation, camptothecin, or etoposide (**Figure 4A**). In response to ionizing radiation, parental V79 cells showed minimal radiosensitization with any of the DDR inhibitors in the tested concentrations of 1 μM, whereas NBS1-deficient cells exhibited significant and large radiosensitization upon ATR inhibition (**Figure 4B**). Following camptothecin exposure, V79 cells were significantly sensitized by inhibition of ATM or ATR, while NBS1-deficient cells were selectively sensitized by ATR inhibition. In contrast, during etoposide treatment, DNA-PK inhibition enhanced cytotoxicity in all cell lines, whereas ATR inhibition resulted in selective sensitization of NBS1-deficient cells. Therefore, ATR inhibition induced selective sensitization in ionizing radiation and etoposide treated NBS1 deficient cells. On the other hand, ATR inhibition induced camptothecin sensitization was not NBS1 status dependent. In addition, the camptothecin sensitivity enhanced by ATM inhibition was dependent on the NBS1 proficiency.

### 3.6 DNA damage responses in the co-treatment of camptothecin or etoposide with ATR inhibitor in NBS1 mutant cells

**Figure 5:**
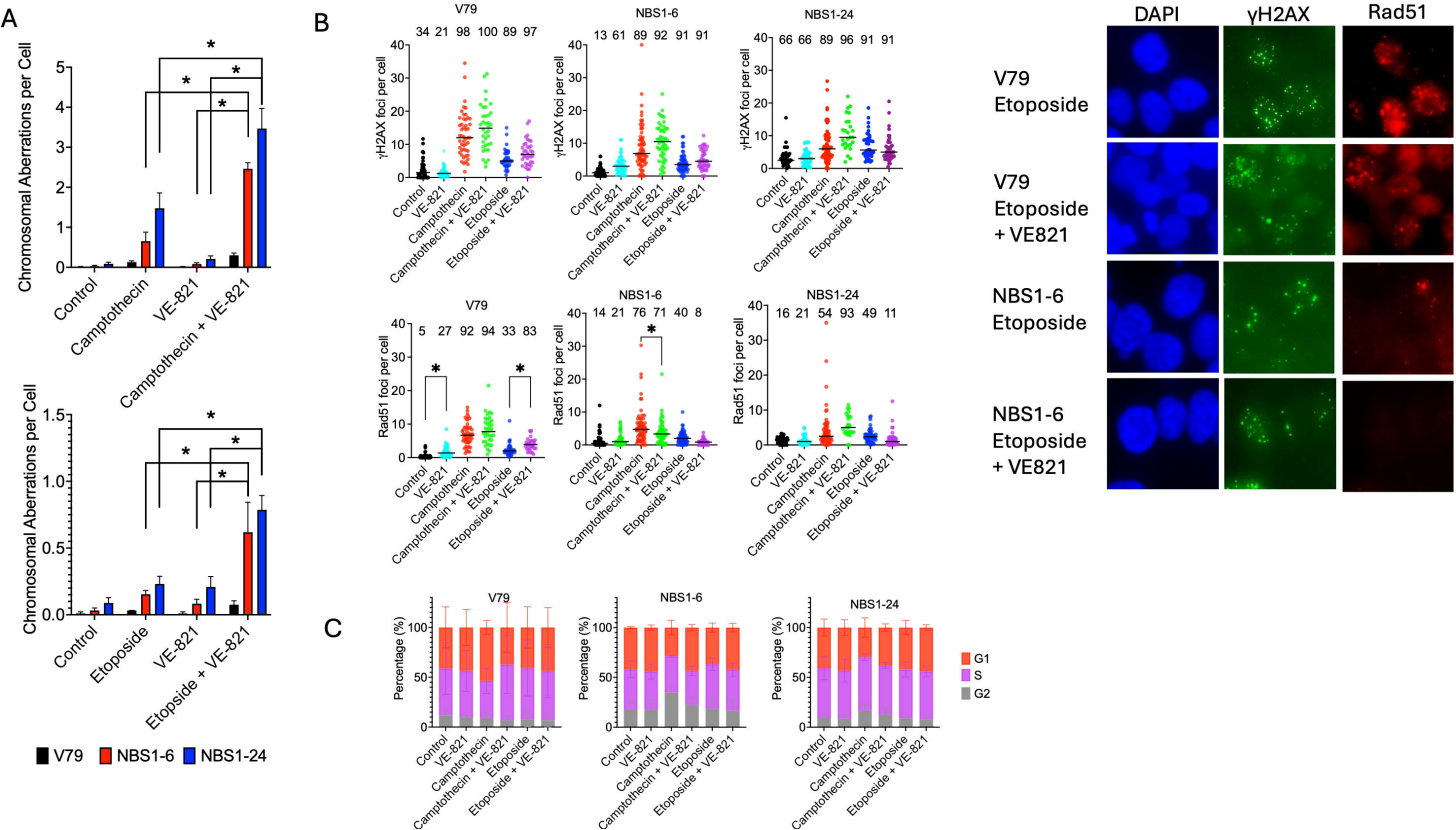
DNA damage responses in the combination of ATR inhibitor and camptothecin or etoposide. **(A)** Chromosome aberration formation, **(B)** γH2AX and rad51 foci formation, Numbers above data indicate percent of cells show foci positive (>3 foci per cell), Representative images of reduced rad51 foci in NBS1–6 cells treated with VE821 and etoposide, **(C)** Flow cytometry analysis after overnight drug treatment. Error bars indicate the standard error of the mean from three independent experiments. *indicates statistical significance (p<0.05, ANOVA).

To validate the elevated cytotoxicity observed with ATR inhibitor treatment in NBS1 mutant cells, chromosomal aberrations were analyzed in synchronized cells (**Figure 5A**). Etoposide and camptothecin were examined at 0–3 hours and 3–6 hours, respectively, to assess G2- and S-phase–specific DNA damage. Both camptothecin and etoposide induced more chromatid breaks in NBS1 mutant cells than in wild-type cells. VE-821 treatment moderately increased chromatid aberrations in mutant cells (approximately 2-fold) but had no effect in wild-type cells. Significant chromosomal aberrations were observed when etoposide or camptothecin was combined with the ATR inhibitor in NBS1 cells.

Further analyzing DNA damage responses, DNA double strand break marker γH2AX and homologous recombination marker Rad51 foci were analyzed following overnight drug treatment (**Figure 5B**). Camptothecin treatment induced a marked increase in γH2AX foci per cell and γH2AX foci positive cells in both V79 and NBS1 mutant cells, regardless of the presence of VE821 treatment. Etoposide treatment did not induce significant increase of γH2AX foci per cell but increased γH2AX foci positive cell fractions. Rad51 foci formation was increased by camptothecin or etoposide treatment as observed per cell and positive cell fractions. However, in the presence of VE-821, NBS1 mutants severely reduced etoposide induced Rad51 foci formation observed as reduced Rad51 foci positive cell fractions. This was not observed in wild type cells.

Drug combination effects were also assessed by flow cytometry (**Figure 5C**). VE-821 did not result in noticeable cell cycle distribution changes. Camptothecin treatment induced moderate but not significant G1 phase accumulation and this was resolved with co-treatment of VE-821 in V79 cells. For both NBS1 mutant cells, camptothecin reduced G1 phase population and increased G2/M population. These alterations were resolved with VE-821 co-treatment. Etoposide and Etoposide plus VE-821 did not present noticeable cell cycle distribution changes.

## 4 Discussion

This study aimed to identify potential novel roles of NBS1 functions in DNA damage responses using Chinese hamster-derived NBS1 mutant cell lines. We successfully generated two novel NBS1-deficient clones using CRISPR/Cas9 technology (**Figure 1**), each harboring distinct mutations. NBS1–6 with a single-base insertion causing a frameshift. NBS1–24 is a hypomorphic mutation with abnormal NBS1 protein expression. Both clones were characterized by their cellular responses to DNA damage. Although the cytotoxicity results agreed between the mutants, DNA damage responses did not agree fully. It suggested NBS1–24 may not be a complete deficiency in the NBS1 functions. Our attempt to detect NBS1 protein expression by Western blotting did not work ideally because the antibody partially recognizes hamster NBS1 protein (**Supplementary Figure 2**). Therefore, we could not fully confirm this. Complete NBS1 knockout *in vivo* is challenging, as NBS1 depletion leads to embryonic lethality in mice (19, 20). In humans, Nijmegen breakage syndrome arises from hypomorphic mutations that typically generate two truncated NBS1 fragments, resulting in reduced, rather than abolished, protein expression (21). *In vitro*, knockout cell lines with hypomorphic mutations have previously been generated *in vitro* using various mammalian cell culture systems (5, 9, 22–25) and in our studies we successfully established NBS1 mutants for the first time in a Chinese hamster system, which offers a strong advantage for cytogenetic analysis. While the establishment of NBS1 mutant lines in Chinese hamster V79 cells provides advantages for cytogenetic analysis due to their stable karyotype and suitability for chromosomal aberration assays, several limitations should be considered. Protein-level characterization in hamster cells can be technically challenging due to the limited availability of well-validated antibodies recognizing hamster proteins. We could detect part of NBS1 protein using a third antibody (**Supplementary Figure 2**). The initial two antibodies Protein Tech rabbit polyclonal 55025-1-AP and Novus rabbit polyclonal NB100-143SS did not work for protein detection at all. Furthermore, as a non-human model, V79 cells do not fully recapitulate the molecular and clinical complexity of Nijmegen breakage syndrome in human cells. Species-specific differences in DNA damage response signaling, protein interactions, and regulatory mechanisms may influence phenotype severity and cellular responses. Therefore, extrapolation of findings from hamster cells to human systems should be performed cautiously.

The frameshift mutation in NBS1–6 introduces an early truncation predicted to disrupt critical functional domains of NBS1, including regions required for MRN complex stability and ATM signaling. NBS1-6 exhibited phenotypes consistent with human NBS patient cells and mouse NBS1 knockouts, including elevated cytotoxicity to DNA damage, impaired G2/M arrest, radiation induced chromosomal breaks, and genomic instability (**Figures 2**, **3**) (26, 27). In contrast, exon skipping and large Cas9 insertion in NBS1–24 likely preserves portions of the N-terminal FHA/BRCT domains while altering downstream structural integrity. Partial retention of functional domains could allow residual MRN complex activity, explaining why NBS1–24 behaves more similarly to wild-type cells in certain repair assays while still displaying growth defects. As a key mediator of DNA double strand break sensing and repair (28), NBS1 deficiency sensitizes cells to ionizing radiation and DNA-damaging agents (4). Indeed, the D_10_ values for our mutants were 5.6 and 4.7 Gy, compared with 8.2 Gy in wild type cells (**Table 1**), mirroring the 2–3-fold radiosensitivity seen in human NBS1-deficient cells (4) and the 3–10-fold increase reported in mouse models (29).

Our NBS1 mutant cell lines displayed pronounced hypersensitivity to camptothecin, VE821 and Olaparib (**Figure 2A**). While increased sensitivity to camptothecin and Olaparib has been previously reported in NBS1 deficient systems (30–32), to our knowledge this is the first demonstration heightened sensitivity to ATR inhibitor in NBS1-mutated cells. The NBS1–6 knockout cells exhibited defective cell cycle regulation, failing to maintain G2 arrest following irradiation (**Figure 3B**) and showed chromosomal aberrations together with delayed chromatin repair kinetics (**Figures 3A, C**). These findings are consistent with prior reports describing prolonged DSB repair defects associated with NBS1 deficiency (33). Although NBS1 has been implicated in sensing UV-induced damage and single-stranded DNA at stalled replication forks (34), neither NBS1 mutant clone exhibited increased sensitivity to bleomycin, UV-C or hydroxyurea under the condition tested, which were exponentially growing populations (**Figure 2A**). One possible explanation is that cell cycle specific effects may have been masked in asynchronous growing populations, and that synchronization may be required to reveal differential sensitivities to these agents. Future studies using synchronized cell populations would clarify this limitation. Indeed, cytogenetic analysis conducted in specific cell cycle population demonstrated clear sensitization to DNA damaging agents in NBS mutant cells (**Figure 2**). With respect to ionizing radiation induced damage response, NBS1–6 knockout cells showed impaired G2/M checkpoint activation at low radiation doses (**Figure 3B**), consistent with prior studies (33, 35), and DSB repair capacity measured by PCC also got impaired. (**Figure 3A**). On the other hand, NBS1–24 with abnormal NBS1 protein size presented wild type like DNA damage responses (**Figures 3A, B**). The difference between the two clones in DNA damage response could be due to the null mutation clone and hypomorphic clone with partially functional protein, which has been described in the majority of known pathogenic hypomorphic variants in Nijmegen breakage syndrome patients (36–38).

Among the three DDR kinase inhibitors tested, ATR inhibition selectively sensitized NBS1 deficient cells, highlighting a compensatory dependence on ATR signaling for survival following DNA damage induced by ionizing radiation (**Figures 4A, B**). To further dissect the cell cycle specific interactions between ATR and NBS1, we compared the effect of the S-phase specific topoisomerase I inhibitor camptothecin with those of the G2 phase biased topoisomerase II inhibitor etoposide (**Figures 4A, B**). ATR inhibition enhanced camptothecin induced cytotoxicity in both wild type and NBS1 deficient cells, consistent with the well established role of ATR in protecting replication forks and coordinating repair during S-phase, independence of NBS1 status. In contrast, etoposide treatment resulted in selective hypersensitization of NBS1-deficient cells upon ATR inhibition, indicating a specific reliance on ATR-mediated repair pathways during G2 when functional NBS1 is absent (**Figure 4A**).

To elucidate the mechanism underlying ATR inhibitor mediated etoposide sensitization in NBS1 deficient cells, we investigated chromosomal aberration, γH2AX and Rad51 foci, and cell cycle progression. ATR inhibition alone moderately increased chromatid breaks in NBS1 mutants, whereas combined ATR inhibitor with camptothecin or etoposide led to a marked increase in chromosomal damage compared with wild-type cells (**Figure 5A**). While γH2AX induction was not selectively affected in NBS1 mutant cells, Rad51 foci formation was strongly suppressed by ATR inhibition following etoposide treatment in NBS1 mutant cells (**Figure 5B**). Cell cycle distributions were not substantially affected by etoposide in the presence of ATR inhibition (**Figure 5C**). Collectively, these results indicate that ATR plays a critical role in supporting homologous recombination repair in NBS1-deficient cells, particularly in response to DSBs arising in G2 phase.

Collectively, our data demonstrate that while ATR participates broadly in DNA damage response, NBS1-deficient cells are specifically dependent on ATR to repair DSBs occurring in G2, whereas S-phase lesions induced by camptothecin are handled by ATR through a pathway that is largely independent of NBS1. This selective reliance on ATR highlights potential synthetic lethal interactions that could be exploited therapeutically in contexts of NBS1 deficiency.

## Data Availability

The original contributions presented in the study are included in the article/**Supplementary Material**. Further inquiries can be directed to the corresponding author.

## Supplementary material

Supplementary Figure 1 RT-PCR analysis of NBS1–24 cells. A) Three amplified transcripts after RT-PCR.

Supplementary Figure 2 Western blot analysis of NBS1 mutated cells. **(A)** NBS1 protein expression in three cell lines.
