# Supplementary figures and images for: Generation of NBS1 knockout in Chinese hamster cells revealed ATR role for radiation and etoposide induced DNA damage in absence of NBS1 proteins
Source: Front Oncol. 2026 Mar 25;16:1776137. doi: 10.3389/fonc.2026.1776137 (PMC13056612; doi:10.3389/fonc.2026.1776137)

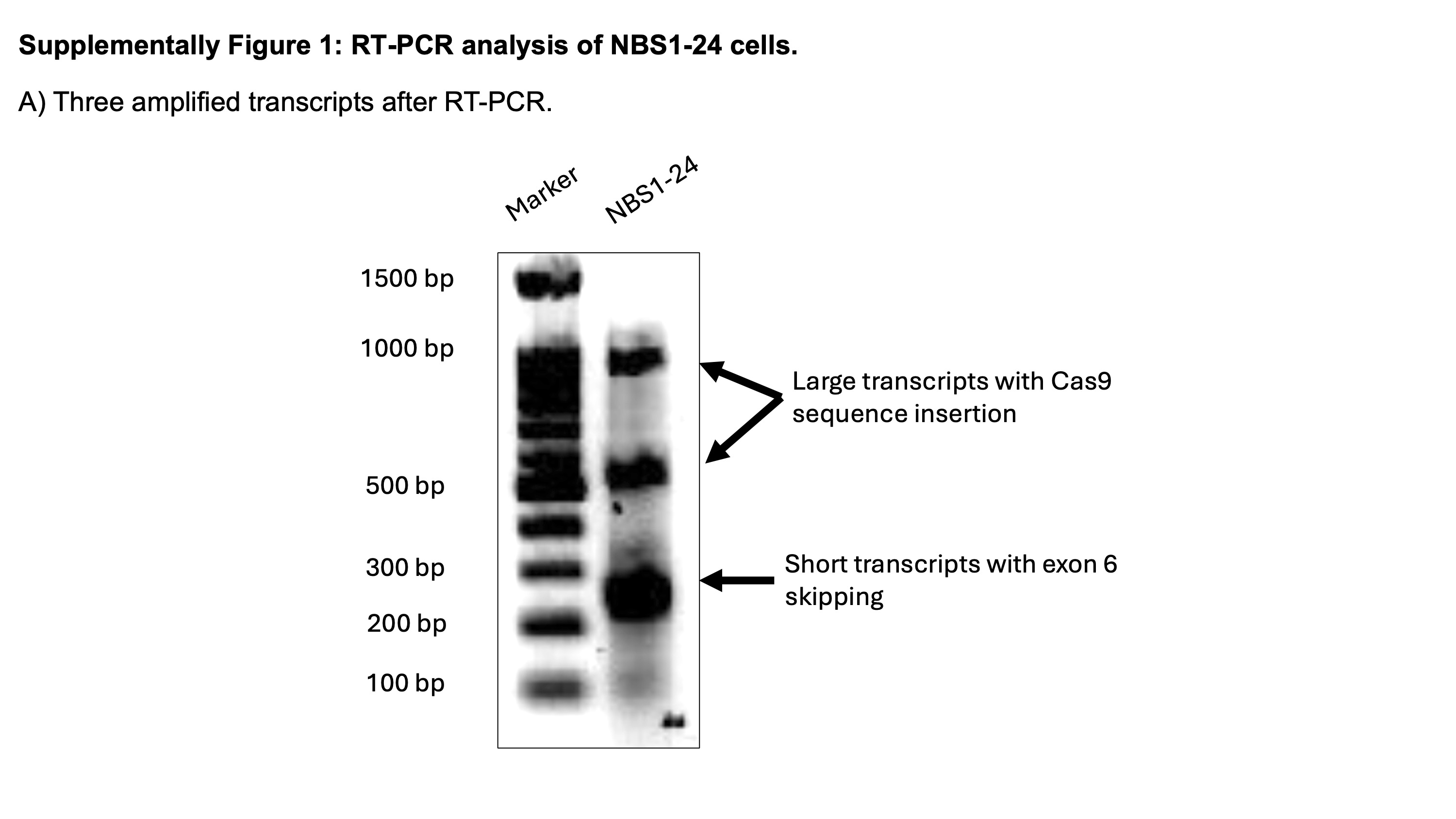

Supplement: Supplementary Figure 1 — RT-PCR analysis of NBS1–24 cells. A) Three amplified transcripts after RT-PCR. [file Image1.jpeg]

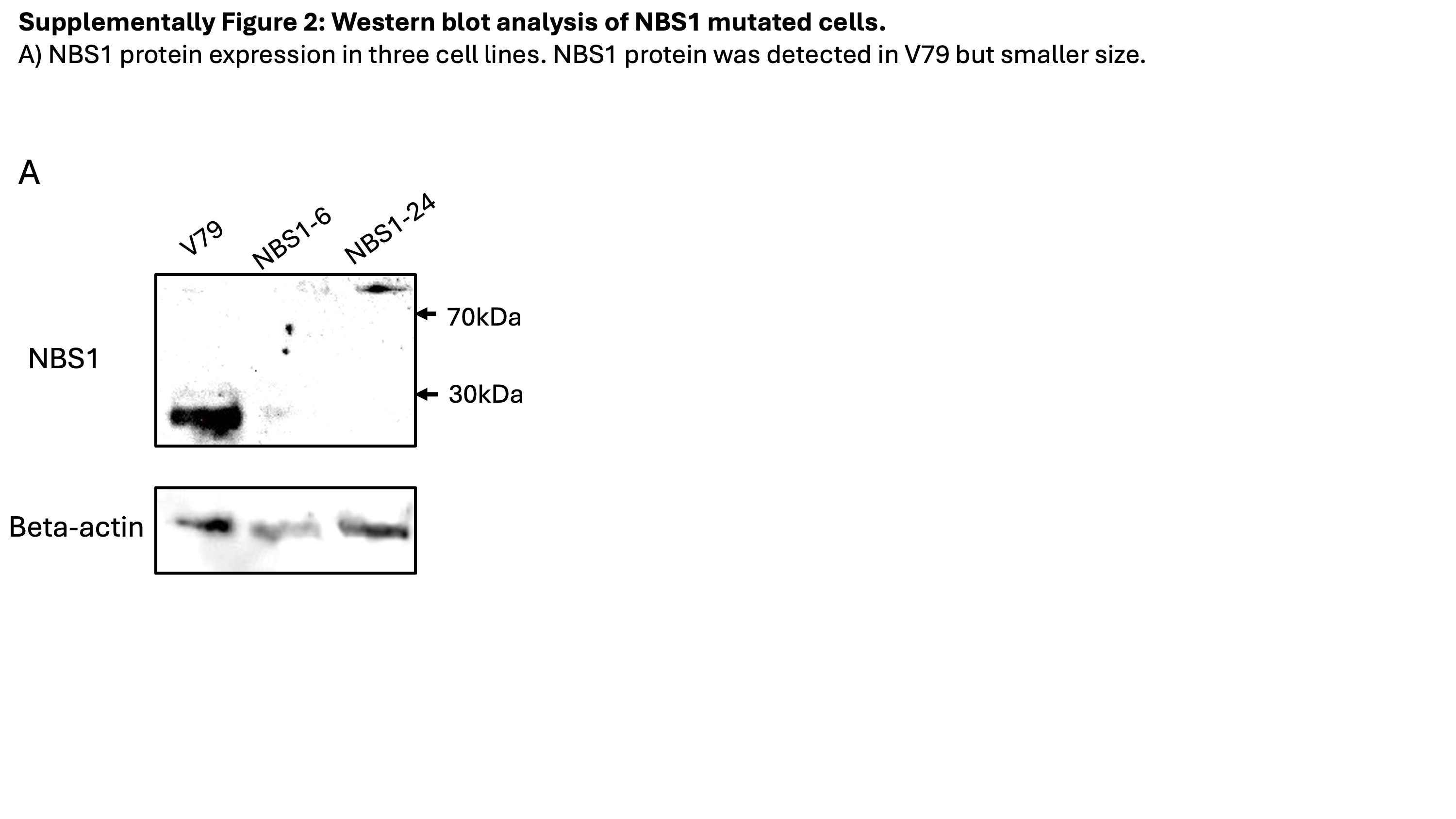

Supplement: Supplementary Figure 2 — Western blot analysis of NBS1 mutated cells. (A) NBS1 protein expression in three cell lines. [file Image2.jpeg]
